# Thermally Induced Structural Transitions of Nylon 4 9 as a New Example of Even–Odd Polyamides

**DOI:** 10.3390/polym10020198

**Published:** 2018-02-16

**Authors:** Cristian Olmo, Maria Teresa Casas, Juan Carlos Martínez, Lourdes Franco, Jordi Puiggalí

**Affiliations:** 1 Chemical Engineering Department, EEBE, Polytechnic University of Catalonia, Building I.2, C/Eduard Maristany 10-14, 08019 Barcelona, Spain; olmocristian@gmail.com (C.O.); m.teresa.casas@upc.edu (M.T.C.); 2 ALBA Synchrotron Light Facility, Carrer de la llum 2-26, Cerdanyola del Vallès, 08290 Barcelona, Spain; guilmar@cells.es; 3 Research Center for Multiscale Science and Engineering, Polytechnic University of Catalonia, C/Eduard Maristany 10-14, 08019 Barcelona, Spain

**Keywords:** polyamides, thermal transitions, hydrogen bonds, lamellar morphology, spherulites, synchrotron radiation, crystalline structure

## Abstract

Crystalline morphology and structure of nylon 4 9 have been studied by means of optical and transmission electron microscopies, and X-ray diffraction. Rhombic crystals were characteristic of crystallization from glycerin dilute solutions, although the final morphology was dependent on the crystallization temperature. In any case, a single electron diffraction pattern was always obtained, being characteristic a 2 *mm* symmetry and reflections at spacings that were indicative of a projected rectangular unit cell with hydrogen bonds established along two planar directions (i.e., the diagonals of the unit cell), as it was determined from related polyamides. Crystallization from the melt gave rise to negative birefringent spherulites with a morphology (axialitic, speckled or ringed) that was dependent on the crystallization temperature. Kinetic analysis indicated that melt crystallization took place according to two growth mechanisms (Regimes II and III), which reflect distinct secondary nucleation rates. A complex polymorphic behavior on heating and cooling processes was evidenced by real time synchrotron experiments, being determined an intermediate crystalline structure as well as the typical pseudohexagonal arrangement associated to the Brill transition. Polymorphic transitions were highly dependent on the initial crystalline structure, being enhanced the structural transition from the low temperature structure to the intermediate one when traces of the latter were initially present. Calorimetric and infrared studies supported also the detected thermal transitions of nylon 4 9.

## 1. Introduction

Aliphatic polyamides (nylons) are the first synthetic and semicrystalline polymers that displayed outstanding mechanical and thermal properties to be used as fibers and engineering thermoplastics [1,2]. Nylons are nowadays employed in a wide range of applications that mainly cover the textile, and automotive industries. The high performance of polyamides is a consequence of the strong intermolecular interactions that can be established between their constitutive NH and CO groups. Nylons are often classified according to the type of monomers implied in the polymerization, being distinguished between those obtained from a single monomer (i.e., AB polymers prepared by ring opening polymerization of lactams or alternatively by condensation of α-amino acids or their derivatives) and those obtained from two monomers (AABB polymers prepared by the polycondensation of a diamine and a dicarboxylic acid or their derivatives). Such polymers are usually named as nylons *n* and nylons *n m*, being *n* and *m* indicative of the number of carbon atoms of the respective monomers. Final properties of nylons are logically dependent on the density of amide groups (i.e., the specific values of *n* and *m*), but also on the crystalline structure. This structure should correspond to a minimum of the packing energy and consequently is governed by the formation of hydrogen bonds, keeping a practically linear geometry, between all constitutive amide groups. In this sense, the parity of the number of carbon atoms of the different monomers (i.e., odd or even *n* and *m* values) plays a remarkable influence on structure and properties. Nylon 6, nylon 11, nylon 12, nylon 6 6, nylon 6 10 and nylon 6 12 are specifically the aliphatic polyamides with higher commercial applications [1].

In general, the structure of such commercial nylons is based on the stack of sheets composed of hydrogen bonded molecular chains that could be in a parallel, antiparallel (e.g., even nylons) or both (e.g., odd nylons) dispositions, and had a practically all trans molecular conformation [3,4]. The antiparallel arrangement was postulated to be preferred for the odd nylons [5], while for centrosymmetric even–even nylons both dispositions are equivalent. Weak van der Waals forces are established between the consecutive hydrogen bonded sheets, being the packing energy optimized by a shift along both the single hydrogen bonding direction and the chain axis direction. This displacement may be progressive or recuperative giving rise to the well-known α and β structures, respectively. The corresponding X-ray fiber diffraction patterns of such crystalline forms are characterized by the presence of two strong equatorial reflections associated with intrasheet and intersheet spacings (at 0.440 and 0.380 nm, respectively) and a meridional or off-meridional orientation of reflections associated to the chain repeat for recuperative and progressive shears, respectively. Experimental limitations of fiber X-ray diffraction analyses (broad and scarce number of reflections) induced to use other approximations to confirm the structural models. Force field calculations [6] and resolution of small model compounds by direct methods [7] can be mentioned.

Even nylons with many carbon atoms (e.g., nylon 12) crystallize according to a pseudohexagonal packing (defined by one or two close equatorial reflections in the 0.420–0.400 nm range), a deviation towards skew conformations for torsional angles of methylene-amide bonds, and a tilting of amide groups from the plane defined by the methylene carbon atoms [8]. In this way, hydrogen bonds become established between parallel chains and along a single direction that becomes outside the sheet plane defined by the methylene groups. This arrangement (γ form) is also found as a polymorphic form of even nylons with a medium length of its polymethylene segment (e.g., nylon 6 [9]), and was also postulated for odd–odd nylons due to impossibility to form a good hydrogen bonding geometry between chains having an all trans conformation [10]. The high applied interest of the indicated conventional polyamides have involved a considerable structural research, which is complex due to the indicated variety of molecular arrangements and molecular conformations that deals to polymorphic structures as it is the case of nylon 11 [11].

Thermal induced structural transitions are also a common characteristic observed for conventional nylons displaying the indicated α/β forms. In this way, equatorial reflections gradually merge into a single peak indicative of a pseudohexagonal arrangement at the named Brill temperature. This transition seems reversible but shows a hysteresis effect since it took place at a lower temperature on cooling. Understanding of Brill transition is problematic and different interpretations have been postulated [12,13,14,15,16,17,18]. In this way, it has been proposed an increased mobility of methylene groups that lead to a pseudohexagonal packing or alternatively the disruption of the initial hydrogen bonds (note that they are established along a single direction for α, β and γ forms) to form new interactions randomly distributed along three planar directions at 120°.

New structures have also been determined for nylons having special units such as monomethylendiamine, malonamide and glycine. In these cases, a single methylene group is placed between two amide groups causing distinctive conformational preferences that lead to structures with a single (nylons 1 *n* [19]), double (nylons *n* 3 [20]) and three (nylons 2/*n* [21]) coplanar hydrogen-bond orientations. Such structures were peculiar and consequently were also corroborated by the study of small model compounds [22,23,24].

Structures having two hydrogen bond orientations have also been postulated for several odd–even (e.g., nylons 5 6 [25], 5 10 [26], 9 2 [27], 11 10 [28], 11 12 [29] and 13 6 [30]) and even–odd (e.g., nylons 6 5 [31], 12 5 [32], 6 13 [33], 4 7 [34], 6 9 [35,36] and 8 9 [37]) nylons having longer polymethylene sequences (i.e., without a single methylene). In this case, new structures were a consequence of the impossibility to establish correct hydrogen bond interactions when odd diamides having extended conformations were involved as will then be explained in more detail. The interest on the study of odd derivatives is nowadays increasing due to two main points: (a) The necessity to produce polymers from renewable resources that could substitute fossil-based materials [38,39,40,41]. In this sense, nylons 5 6 [42,43,44,45], 6 9 [46] and 13 6 [30] are gaining attention. (b) The odd numbered polyamides may display ferroelectric properties [47,48] and in particular the peculiar structure of nylon 6 9 has been considered to get polymers with piezoelectric activity [46].

The structural versatility of polyamides gave rise to a wide variety of spherulitic morphologies for a given polymer, being still unclear the drastic change with crystallization temperature of physical properties such as the birefringence sign. Therefore, it has been postulated that lamellar crystal growth inside spherulites varied and specifically conventional nylons gave rise to positive or negative spherulites depending if hydrogen bonds had a radial or tangential orientation, respectively [49,50]. Obviously, complexity increased for structures having two hydrogen bond orientations as it is the case of odd derivatives [51]. Lately, efforts have been focused to relate morphological evidence with structural changes induced by temperature for different even–odd polyamides (i.e., nylons 4 7 [34] and 6 9 [36]). Interestingly, properties were dependent on the edge-on and flat-on disposition of constitutive lamellae, displaying in the last case reversible changes on birefringence with temperature as a consequence of polymorphic transitions.

Nylon 4 9 corresponds to a scarcely studied even–odd polyamide that has an intermediate composition of those recently considered (i.e., the total number of carbon atoms is 13 instead of 11 (nylon 4 7) or 15 (nylon 6 9)). The present work is focused on the comprehension of four aspects: (a) characterization of the low temperature predominant form from the study of structure and morphology of single crystals; (b) study of thermally induced phase transformations on heating and cooling processes by means of real time synchrotron experiments and having special consideration on the occurrence of intermediate crystalline structures; (c) evaluation of the influence of the sample preparation process on the crystalline structure and the derived thermal induced structural transitions; and (d) study of spherulites developed at different crystallization temperatures, considering morphology, properties and crystallization kinetics.

## 2. Experimental Section

### 2.1. Materials and Synthesis of Nylon 4 9

All reagents and solvents were purchased from Sigma-Aldrich (Sant Louis, MO, USA) and used without purification. Nylons 4 9 was synthesized by interfacial polycondensation of azeloyl dichloride with 1,4-diaminobutane. To this end, 15 mmol of the dichloride were dissolved in 75 mL of dry carbon tetrachloride and vigorously stirred while 75 mL of an aqueous solution containing 35 mmol of 1,4-diaminobutane and 6.4 g of sodium carbonate was dropwise added. After addition was complete, stirring was kept for 30 min and then the powdered polymer was recovered by filtration, washed successively with water, ethanol and ethyl ether before drying in a vacuum desiccator at 60 °C. After that, it was purified by precipitation, by adding water to a dilute formic acid solution.

### 2.2. Measurements

Molecular weights of synthesized samples were determined by gel permeation chromatography (GPC). A Shimadzu LC-8A cromatograph (Shimadzu, Tokyo, Japan) equipped with an Empower computer program (Waters) was employed together with a PL HFIP gel column (Polymer Lab, Agilent Technologies Deutschland GmbH, Böbligen, Germany)) and a refractive index detector RID-10A (Shimadzu, Tokyo, Japan). Molecular weights were calibrated using polymethyl methacrylate standards.

Infrared absorption spectroscopic data were acquired with a FTIR 4100 Jasco spectrometer (Jasco International Co. Ltd., Tokyo, Japan) dotted with attenuated total reflection (ATR) (Specac model MKII Golden Gate. A heated Diamond Top-Plate was employed to study thermal induced transition.

^1^H nuclear magnetic resonance (NMR) spectra were recorded on A Bruker AMX-300 spectrometer (Bruker Co., Bremen, Germany) at 25.0 °C operating at 300.1 MHz and 25 °C was used to record ^1^H NMM spectra. A mixture of deuterated chloroform and trifluoroacetic acid (9:1) was employed as solvent. Tetramethylsilan was added as internal reference.

Differential scanning calorimetric data were recorded with a TA instrument Q100 series (New Casttle, DE, USA) equipped with a refrigerated cooling system. Measurements were carried out with samples weighting approximately 5 mg and under a flow of dry nitrogen. Thermal characterization involved: a first heating run (20 °C/min) of the as-synthesized sample, a cooling run (10 °C/min) after keeping the sample in the melt state for 3 min to erase thermal history and a subsequent heating run (20 °C/min) to determine the behavior of a non-isothermally crystallized sample.

Thermogravimetric (TGA) and differential thermogravimetric (DTGA) data were acquired with a Q50 thermogravimetric analyzer of TA Instruments (New Casttle, DE, USA) under a flow of dry nitrogen with approximately 5 mg samples and at a heating rate of 20 °C/min.

Samples for optical microscopy observations were prepared from small sections of melt-crystallized films, which were subsequently pressed between cover slides, inserted in a hot stage, heated a 10 °C higher than its melting point for 3 min and quickly cooled to the selected isothermal crystallization temperature. A Zeiss Axioscop 40 Pol light polarizing microscope (Carl Zeiss, Göttingen, Germany) was employed to measure the spherulite groth rate, while temperature was controlled with a Linkam system having a THMS 600 heating and freezing stage connected to an LNP 94 liquid nitrogen cooling system (Linkam Scientific, Tadworth, UK). A Zeiss AxiosCam MRC5 digital camera (Carl Zeiss, Göttingen, Germany) was employed to get micrographs at appropriate time intervals. Sign of spherulite birefringence was determined inserting a first-order red tint plate between crossed polarizers.

Lamellar crystals of nylon 4 9 were obtained by isothermal crystallization in dilute (ca. 0.1 mg/mL) glycerin solutions at temperatures between 90 and 130 °C. In all cases, the crystals were recovered from the mother liquor by centrifugation, repeatedly washed with *n*-butanol and deposited on carbon-coated grids, which were shadowed with Pt–Carbon at an angle of 15° for bright field observations.

A Philips TECNAI 10 electron microscope (Philips Electron Optics, Eindhoven, The Netherlands) was used and operated at 80 and 100 kV for bright field and electron diffraction modes, respectively. Selected area electron diffraction patterns and bright field micrographs were taken with a SIS MegaView digital camera (Olympus Soft Imaging Systems Inc., LLC, Lakewood, WA, USA). Diffraction patterns were internally calibrated with gold (d_111_ = 0.235 nm).

The real-time variable temperature synchrotron study was carried out on beamline BL11-NCD at ALBA (Cerdanyola del Vallès, Barcelona, Spain) by using a wavelength of 0.100 nm and a WAXD LX255-HS detector from Rayonix. Polymer samples were confined between Kapton films and then held on a Linkam hot stage with temperature control within ± 0.1 °C. WAXD profiles were acquired during heating and cooling runs in time frames of 20 s and rates of 10 °C/min. WAXD diffraction patterns were calibrated by means of a geometrical calibration process of a well-known sample (standard Cr_2_O_3_). Diffraction profiles were normalized to the beam intensity and corrected considering the empty sample background.

## 3. Results and Discussion

### 3.1. Chemical Characterization

**Figure 1:**
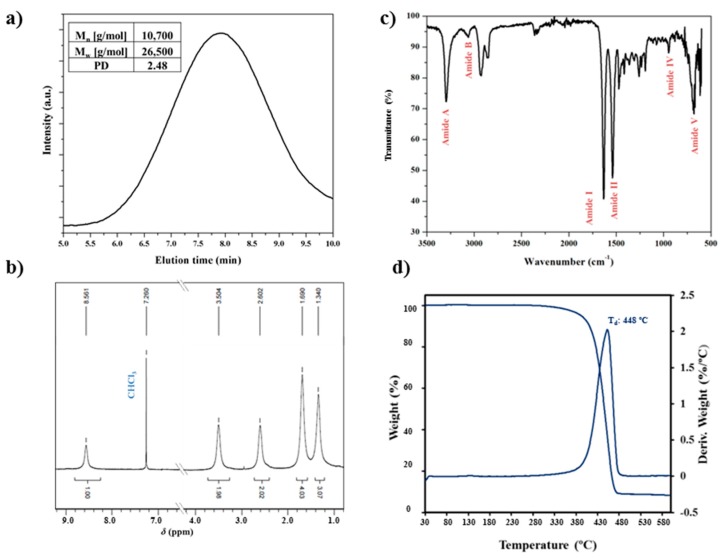
GPC chromatograph (**a**); FTIR spectrum (**b**); ^1^H NMR spectrum (**c**); and TGA/DTGA thermogravimetric curves (**d**) of synthesized nylon 4 9.

Nylon 4 9 was obtained with a yield of 60%, polydispersity index of 2.5 and number average molecular weight of 21,000, as determined by GPC (Figure 1a). Index was slightly higher than theoretically expected from Flory’s theory (i.e., 2.5 with respect to 2.0), suggesting the presence of a small ratio of oligomers. FTIR and ^1^H NMR spectra of the synthesized nylon are given in Figure 1b,c, respectively. Spectra were fully consistent with the anticipated chemical constitution. Specifically, infrared spectra showed the characteristic absorption bands of amide and methylene groups at: ≈3291 cm^−1^ (Amide A, N-H stretching), ≈3066 cm^−1^ (Amide B, overtone of Amide II), 2927 and 2851 cm^−1^ (asymmetric and symmetric CH-stretching bands), ≈1633 cm^−1^ (Amide I, C=O stretching), ≈1539 cm^−1^ (Amide II, C-N stretching and CO-N-H bending), ≈940 cm^−1^ (Amide IV, C-CO stretch) ≈721 cm^−1^ (CH_2_ waging), and ≈684 cm^−1^ (Amide V, N-H out of plane bending). ^1^H NMR spectra were characterized by peaks at: 8.56 ppm (NH, broad, 2H), 3.50 ppm (NHC*H*_2_, broad, 4H), 2.60 ppm (COCH_2_, triplet, 4H), 1.69 ppm (NHCH_2_C*H*_2_, broad, 4H + COCH_2_C*H*_2_, broad, 4H) and 1.34 ppm (-(CH_2_)_3_-, broad, 6H). Nylon 4 9 had a high thermal stability as demonstrated by the thermogravimetric curves displayed in Figure 1d. The onset degradation temperature was close to 290 °C, being the decomposition process defined by a single step with a maximum DTGA peak at 448 °C. This stability appears highly important to discard any evidence of thermal degradation during the heating scans at which samples were submitted for the study of structural transitions.

### 3.2. Morphology of Nylon 4 9 Single Crystals

**Figure 2:**
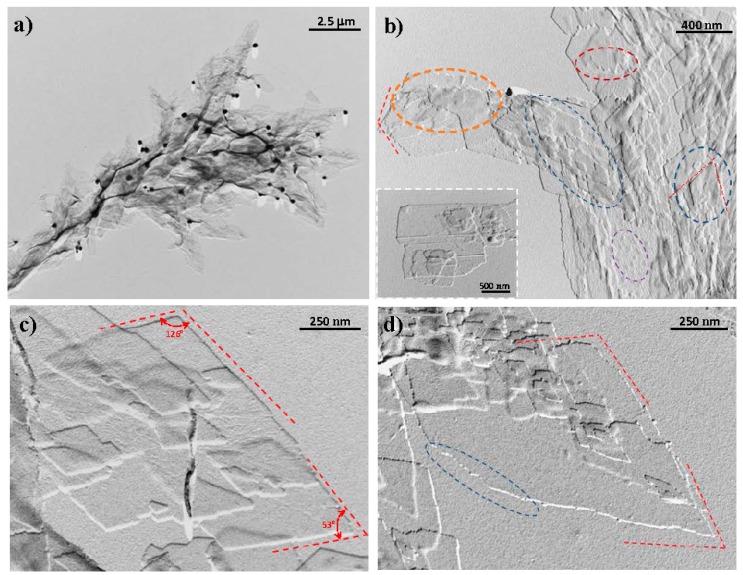
Transmission electron micrographs of nylon 4 9 crystals obtained from dilute glycerin solutions, illustrating the influence of crystallization conditions on morphology: (**a**) Arm of an spherulitic aggregate obtained at 90 °C. (**b**) Aggregates constituted by planar crystals obtained at 110 °C. Blue ellipsoid shows the presence of rhombic crystals whereas the orange ellipsoid and the inset point out the presence of lath shaped crystals. Serrated faces and globules are marked with garnet and violet ellipsoids, respectively. (**c**) Rhombic lamellar crystals obtained from crystallizations performed at 120 °C. Red dashed lines define the theoretical angles between growth faces. (**d**) Elongated lamellar morphologies obtained at 130 °C. Dashed lines indicate the theoretical growth faces according to the expected rhombic morphology. Obtuse angles could be recognized although curved morphologies could also be detected (blue ellipsoid).

Crystals suitable for electron microscopy studies were obtained by isothermal crystallization from dilute solutions in glycerin. Their morphology varied depending on the conditions used for the preparation of samples and specifically on the selected temperature. Thus, crystallizations performed at temperatures higher than 90 °C rendered aggregates derived from a common nucleus having radial arms with well-defined extremities (Figure 2a). Crystals with different orientations could be detected (i.e., parallel, tilted and perpendicular to the film surface).

By contrast, planar aggregate structures were observed at temperatures close to 110 °C. The morphology of constitutive lamellae was highly variable although rhombic crystals were predominant over lath shaped crystals (Figure 2b). Different irregularities were common and easily detected, such as the presence of highly serrated edges and even of striations and globules on the surface of crystals. The best lamellar crystals were attained at 120 °C (Figure 2c). An obtuse angle of 12° was measured accurately while the acute angle was less well defined (ca. 50°–53°), and specifically the rhombic appearance was lost at higher temperatures (e.g., Figure 2d for crystallization performed at 130 °C). Obtuse angles could be well recognized, whereas the elongated morphology gave rise to a significant deviation of the acute angle. In all cases, individual lamellae were about 9 nm thick, as estimated from their shadows in the micrographs. This low thickness value and the high molecular weight of the polymer clearly indicate that molecular chains were folded, as is well established for polymer lamellar crystals.

**Figure 3:**
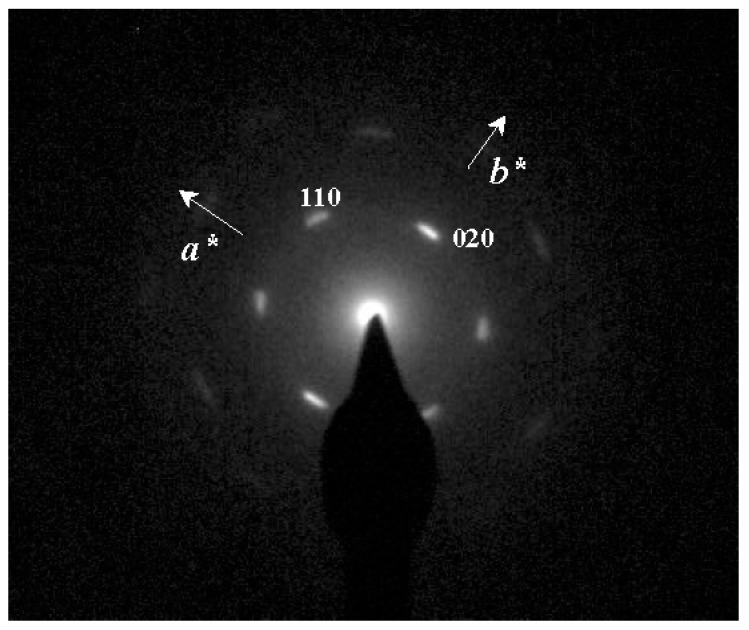
(**a**) Selected-area electron diffraction pattern of nylon 4 9 single crystals prepared from glycerin at 120 °C. Only the most intense *hk*0 reflections are labeled. The pattern shows a clear 2 mm symmetry and reciprocal axes are labeled. (**b**) X-ray diffraction profile of a nylon 4 9 sample isothermally crystallized from diluted glycerin solution.

Electron diffraction patterns of the different nylon 4 9 crystals have the same features, indicating that a single structure was always obtained. However, crystals prepared from glycerin at 120 °C gave the best pattern (Figure 3) because single crystals of adequate dimensions could be selected for diffraction. This pattern was characterized by a 2 mm symmetry with four and two prominent reflections at 0.385 and 0.430 nm, respectively. The above symmetry was always observed, but no additional reflections with a variable intensity that could be associated with a twinned structure were detected, as is common in conventional nylons. Pattern resolution was up to 0.160 nm (i.e., 150 reflection) and allowed a rectangular unit cell with *a* and *b* dimensions of 0.430 nm and 0.860 nm, respectively, to be determined. The angles between the indexed 110 and −110 reflections and between 110 and 1–10 reflections were 126° and 54°, respectively, in full agreement with the obtuse and acute angles observed in the bright field micrographs. Therefore, the lateral faces of rhombic crystals corresponded to 110 planes.

Diffraction data of solution crystallized nylon 4 9 samples agree with previous observations performed with different odd–even [25,26,27,28,29,30] and even–odd [3,4,5,6,7,8,9,10,11,12,13,14,15,16,17,18,19,20,21,22,23,24,25,26,27,28,29,30,31,32,33,34,35,36,37] nylons. In all these cases, hydrogen bonds were not well established between molecular chains having an extended conformation and along a single direction, as is typical of the sheet structure described for conventional nylons. Therefore, a different molecular arrangement involving the establishment of intermolecular hydrogen bonding interactions along two directions was postulated because it could justify the observed symmetry of the electron diffraction pattern. This structure implied minimum distortion of the zig-zag molecular conformation and was compatible with spacings similar to those reported for conventional α/β forms of conventional nylons.

**Figure 4:**
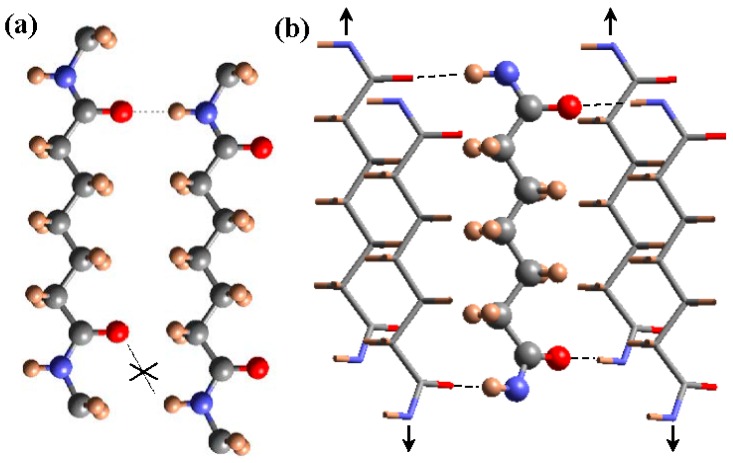
Scheme of the unfavorable hydrogen bonding geometry between odd carboxamide (i.e., pimelamide) units having an all-trans conformation (**a**); and the favorable interaction established according to the proposed structure where hydrogen bonds are established along two directions (**b**). For shake of clarity different representations are employed for the external and inner chains of the unit cell and a shorter dicarboxylic unit (i.e., pimelamide) has been considered. Arrows indicate the shift of neighboring chains with respect the central one. Color code: nitrogen, blue; oxygen, red; carbon, gray; hydrogen, brown. Reproduced with permission from [36], copyright 2015 Elsevier.

Basically, the model is based on a slight deviation towards 150° (or −150°) of the two torsional angles vicinal to the odd diamide units, which allows all NH and CO groups of neighboring chains to be faced. The two amide groups of the odd unit rotated in opposite senses from the plane defined by the methylene carbon atoms, allowing the establishment of a good hydrogen-bonding geometry when neighboring chains became conveniently shifted along the chain axis direction (Figure 4). A monoclinic unit cell containing two molecular segments was derived and the chain axis projection corresponded to a rectangular unit cell, with the dimensions of the diagonals being in full agreement with the expected distances between hydrogen bonded chains. Nylon 9 2 is probably the clearest example of such structure due to its highly rich diffraction pattern and the existence of a highly rigid oxalamide unit in the molecular chain [32].

**Figure 5:**
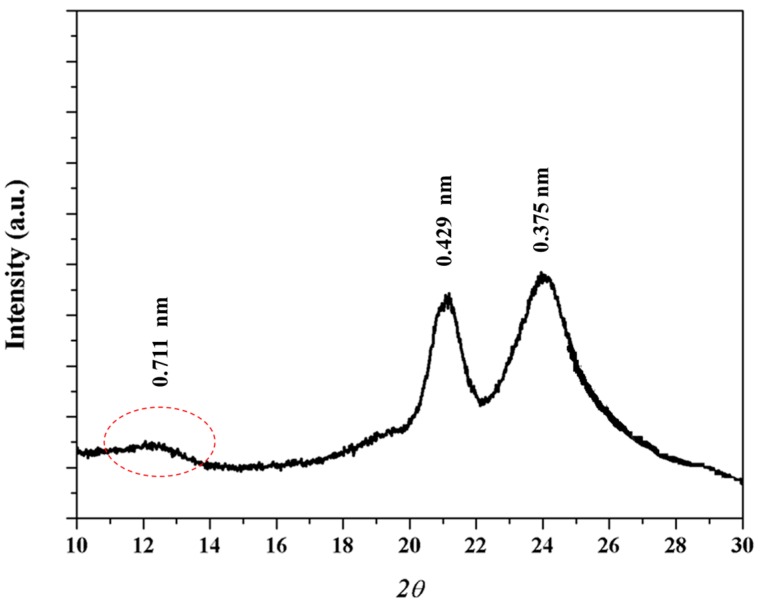
X-ray diffraction profile of as synthesized nylon 4 9 sample.

X-ray diffraction pattern (Figure 5) of the as synthesized sample showed two strong reflections at 0.429 and 0.375 nm that are associated to the molecular packing and correspond with those observed in the electron diffraction patterns. A 002 reflection at 0.711 nm (red ellipsoid) can also be detected and allows postulating a non-orthorhombic structure since this value is clearly lower than the calculated value for the chain repeat length from either an all trans conformation or a typical γ form conformation (0.890 and 0.850 nm, respectively).

### 3.3. Structural Transitions of Nylon 49 during Heating and Cooling Processes from Real Time WAXD Data

**Figure 6:**
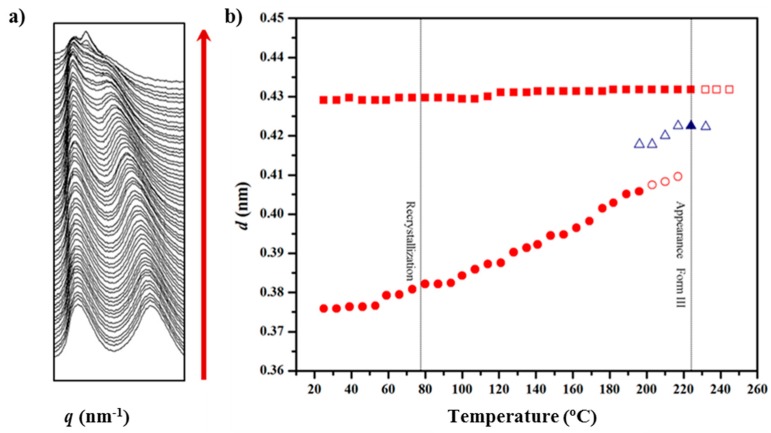
(**a**) Three-dimensional representation of WAXD profiles of a solution crystallized nylon 4 9 sample during heating (10 °C/min) from room temperature to fusion; and (**b**) evolution of the spacings of the two main reflections during the first heating. Full and empty symbols indicate well-defined and intuited reflections, respectively. The temperatures at which structural transitions occur are indicated with vertical lines. Reflections corresponding to Forms I and III are indicated in red and blue, respectively.

**Figure 7:**
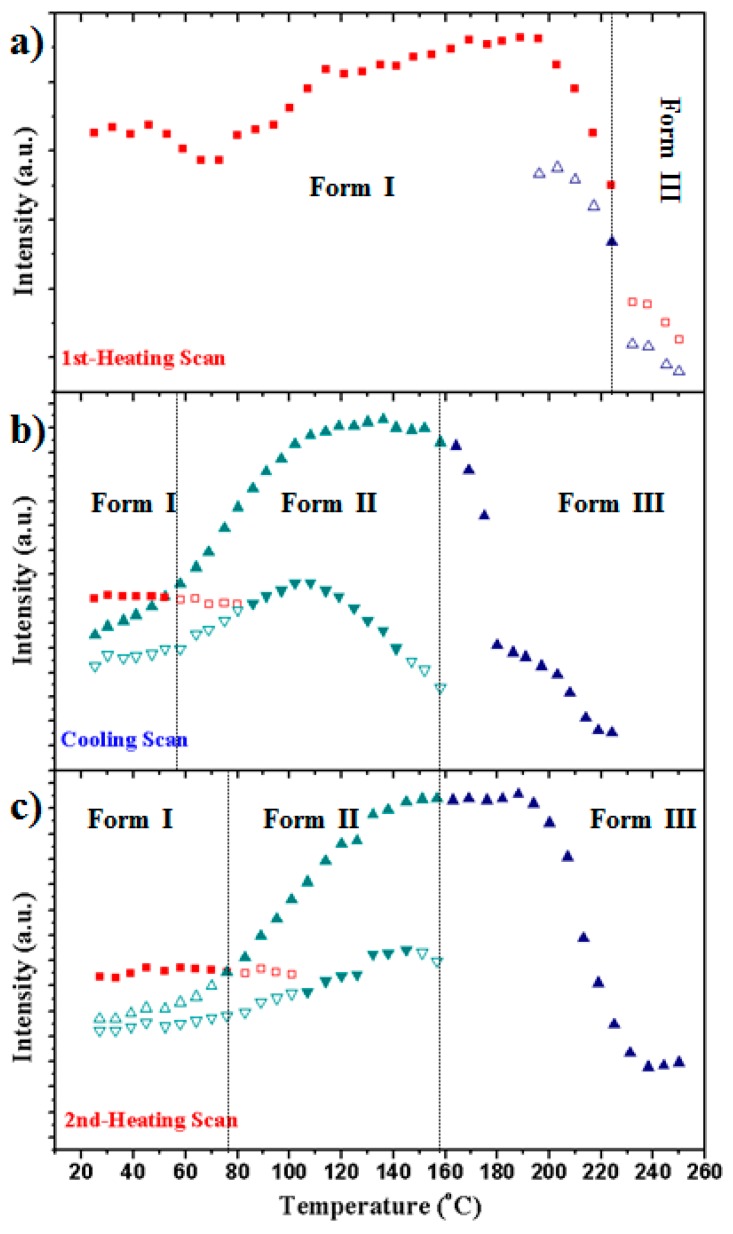
Temperature evolution of the intensity of the main peaks (for the clarity of representation the Form I peak at 0.375–0.410 nm is not plotted) during: the first heating (**a**); cooling (**b**); and second heating (**c**) processes. Full and empty symbols indicate well-defined and intuited reflections, respectively. The temperatures at which structural transitions occur are indicated with vertical lines. Reflections corresponding to Forms I, II and III are indicated in red, green and blue, respectively.

The evolution of the X-ray diffraction pattern with increasing temperature is illustrated in Figure 6a for the two main peaks associated with the molecular packing. The sample recovered from synthesis displayed clear and well-distinguished peaks at 0.430 nm and 0.375 nm, which correspond to the same crystalline form (named Form I), determined for the solution crystallized lamellar crystals. Minor differences are found for the spacing of the 110 reflection, which could be due to the different temperatures at which the crystalline structure was formed (i.e., 25 °C and 120 °C for precipitated and solution crystallized samples, respectively). Temperature evolution of diffraction profiles showed several remarkable features:

(a) The peak at 0.375 nm progressively shifted to higher spacings up to a maximum value of 0.410 nm (Figure 6b), whereas the spacing of the 020 reflection remained practically constant (i.e., 0.430 nm). Basically, the *a* parameter of the unit cell increased (i.e., from 4.30 to 4.67 nm) while the *b* parameter was constant. The projected unit cell therefore became distorted with increasing temperature, with a dilatation being observed in the (100) direction where neighboring chains were closer (i.e., 4.30 nm as opposed to 0.480 nm in the (110) direction). This change can be produced by a small change in the rotation angle between consecutive amide planes (i.e., from 53° to 57°, as determined from basic geometry considerations). It should also be pointed out that the 110 spacing becomes 0.385 at temperatures close to 120 °C, a feature that is in full agreement with the electron diffraction data from lamellae crystallized at such temperature.
(b) Around 120 °C, a recrystallization phenomenon involving Form I can be detected (Figure 6a). This feature is clearer in Figure 7a, where the intensity of mean peak is displayed (see ellipsoid).
(c) At temperatures close to 195 °C a new peak started to appear and reached its maximum intensity just before polymer melting. The spacing of the new reflection increased slightly from 0.418 to 0.423 nm and suggested the formation of the typical high temperature pseudohexagonal packing (named here Form III) that is achieved after the Brill transition. It is interesting that Form I reflections did not meet at high temperature, as is usually observed for conventional nylons. Profiles clearly suggest that Form I progressively disappeared and became the new Form III (see the intensities displayed in Figure 7a). Nevertheless, the transition is not completely clear due to the start of the melting process (225 °C), just when the Form III peak appears better defined.

**Figure 8:**
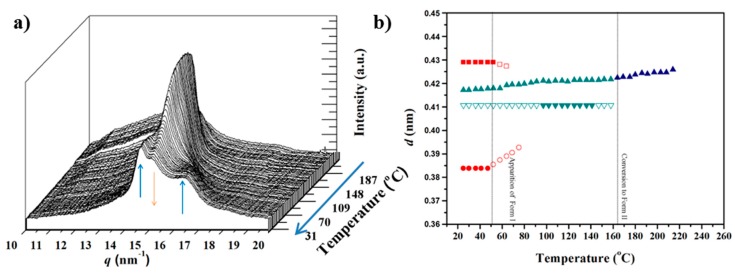
(**a**) Three-dimensional representation of WAXD profiles of nylon 4 9 during cooling (10 °C/min) from the melt to room temperature; and (**b**) evolution of the spacings of the two main reflections during the cooling run. Full and empty symbols indicate well-defined and intuited reflections, respectively. The temperatures at which structural transitions occur are indicated with vertical lines. Reflections corresponding to Forms I, II and III are indicated in red, green and blue, respectively.

The evolution of X-ray diffraction profiles during cooling from the melt state is considerably more complicated because a new crystalline form (named Form II) appeared, as shown in the 3D profiles. The plot of spacings associated with the main observed reflections and the plot of intensity of representative reflections of each crystalline form, as shown in Figure 8a,b and Figure 7b, respectively.

The main features observed in the cooling process can be summarized as follows:

(a) The sample crystallized from the melt in the high temperature Form III, giving rise to a single peak at around 238 °C, which spacing slightly decreased from 0.426 to 0.422 nm when temperature diminished from 230 to 162 °C. This change corresponds to a simple cell contraction and is a reversible process with respect to the observed one during heating. The peak reached its maximum intensity around 162 °C, suggesting that the primary crystallization process completely finished, which is in agreement with calorimetric (at the same cooling rate of 10 °C/min) and optical microscopy observations as then will be shown. Furthermore, diffraction profiles supported, as also will be indicated, that the different kinds of spherulites formed in the 238–229 °C temperature range had the same Form III structure at the temperature where the micrographs were taken (i.e., when further structural transitions that took place on cooling were avoided).
(b) A peak shoulder around 0.410 nm could be envisaged when temperature decreased from 160 °C, being clearly defined at 140 °C. The splitting of the single reflection suggests a minor structural change since a pseudohexagonal packing is still detected (i.e., reflections appeared at 0.420–0.417 nm and 0.410 nm while a single reflection at 0.415 nm is expected for a hexagonal packing defined by a typical hydrogen bonding distance of 0.479 nm between neighboring chains).
(c) A dramatic decrease of the intensity of the two reflections associated to Form II is observed in the 130–60 °C temperature range while new reflections associated to Form I appeared. Profiles clearly demonstrated that a structural transition took place rather than a continuous divergence of reflections as commonly described for a Brill transition. Finally, and with respect to Form I reflections, it can be pointed out that the 110 reflection was again the most sensitive to the temperature change since its spacing decreased from 0.393 to 0.385 nm (a value that is in full agreement with electron diffraction patterns). Logically, structural transition was stopped when the glass transition temperature was reached (i.e., 60 °C) and consequently no further changes on the diffraction profiles were detected.

**Figure 9:**
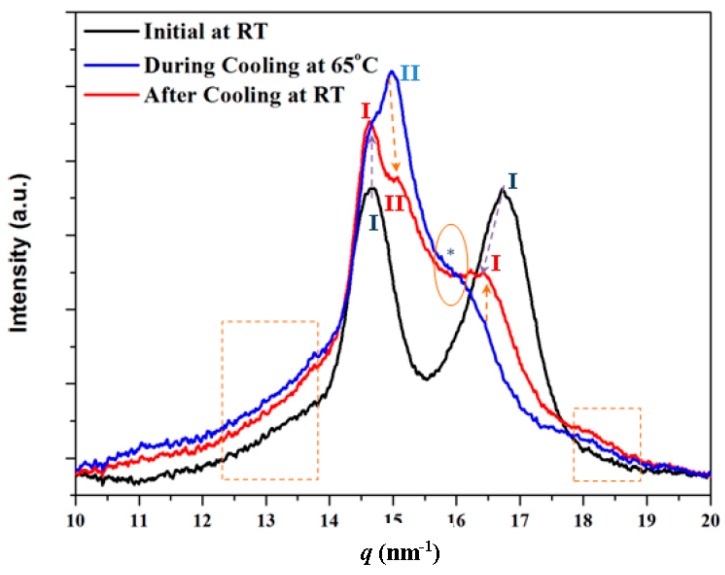
Diffraction profiles taken at room temperature for the solution (black) and melt (red) crystallized samples. Purple arrows compare the peaks associated with Form I. For the sake of completeness, a profile taken during cooling at 65 °C (blue) is also given. Evolution during cooling of some Form I and Form II representative peaks is indicated by the orange arrows and the shoulder associated with Form II with the asterisk and the ellipsoid. Dashed rectangles indicate the regions where tails of the amorphous halo could be detected.

Figure 9 compares the diffraction profiles of nylon 4 9 taken at room temperature for the solution and melt crystallized samples. In the first case, only the two characteristic peaks at 0.430 nm and 0.375 nm were detected with similar intensity. On the other hand, the second peak shifted to 0.385 nm while an additional highly intense peak at 0.417 nm appeared for the melt crystallized sample. The relative intensity between the two peaks associated with Form I also appeared clearly different in the profile of the melt crystallized sample because of the presence of the predominant peak of Form II. A profile taken during the cooling process is also given to emphasize the increase of Form I and the decrease of Form II during cooling, as well as the problematic presence of the shoulder associated with Form II (see ellipsoid and asterisk). Profiles also indicate the presence of an amorphous halo, which appears less intense in the solution crystallized sample and reflects the higher crystallinity of this sample (i.e., see dashed rectangles).

**Figure 10:**
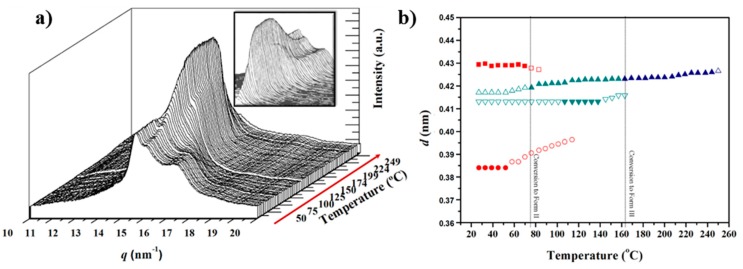
(**a**) Three-dimensional representation of WAXD profiles of a melt crystallized nylon 4 9 sample during the second heating (10 °C/min) from room temperature to fusion. Insets show a different orientation to clarify the temperature evolution of the main reflections. (**b**) Evolution of the spacings of the main reflections during the cooling run. Full and empty symbols indicate well-defined and intuited reflections, respectively. The temperatures at which structural transitions occur are indicated with vertical lines. Reflections corresponding to Forms I, II and III are indicated in red, green and blue, respectively.

Figure 10a,b shows the evolution of profiles and main spacing of a melt crystallized sample during heating. In this case, it is clear that Form I experiences a structural transition towards Form II in the interval temperature between 75 and 120 °C. This behavior clearly differs to the observed one during the first heating run, where Form I is kept up to fusion. It seems that the initial presence of Form II facilitates the polymorphic transition. That is to say, the initial Form II domains displayed a nucleating effect that facilitated the development of new Form II crystals. The plot showing the evolution of spacings (Figure 10b) is similar to that obtained during the cooling run (Figure 8b) although the transition between Forms II and I took place at slightly higher temperatures. It should also be pointed out that the maximum peak intensity during heating processes is observed at a temperature of 185–190 °C, but it corresponds to different crystalline structures: Form I in the first heating run and Form III in the second one. This feature is again a clear indication of the dependence of the structural transition with the distribution of crystalline phases in the initial sample as then will be more extensively discussed.

### 3.4. FTIR Changes during Heating Processes

**Figure 11:**
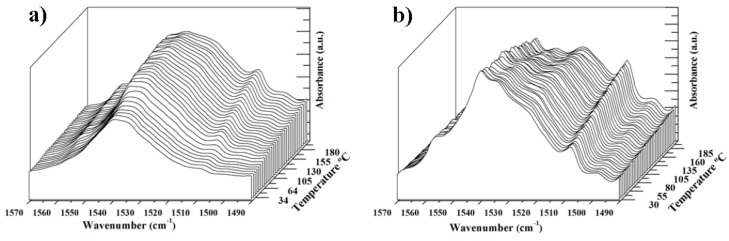
Temperature evolution of the 1570–1485 cm^−1^ region of the FTIR spectra of: solution crystallized (**a**); and melt crystallized (**b**) nylon 4 9 samples.

FTIR spectra are sensitive to structural changes. Specifically, the region between 1560 and 1500 cm^−1^ was found to be very useful to follow the polymorphism of nylon 4 9. The spectrum of the solution crystallized sample showed two minor bands at 1576 cm^−1^ and 1505 cm^−1^, together with a very broad band centered at 1535 cm^−1^, which is typically associated with the Amide II band (coupling of the C-N stretching mode with N-H in plane bending mode) (Figure 11a). The intensity of all these bands increased on heating around a temperature of 95 °C as a consequence of the previously described cold crystallization process. The increase of the intensity of the Amide II band, together with temperature induced broadening, allowed several shoulders to be detected. Nevertheless, no remarkable change was observed until a temperature of 200 °C was reached, which was the maximum value that could be attained with the heater controller adapted to the ATR cell. Therefore, it can be concluded that no structural transition took place, in agreement with synchrotron data.

The sample was subsequently melted outside the ATR cell and then cooled at a rate of 10 °C/min. The room temperature spectrum was well differentiated from those previously described since the Amide II band was clearly split, with two additional peaks appearing at 1540 cm^−1^ and 1524 cm^−1^ (Figure 11b). These peaks could be associated with Form II and had an increased intensity at high temperature while the intensity of the intermediate peak at 1534 cm^−1^ clearly increased during cooling, as expected for Form I. The temperature evolution of FTIR spectra indicates, again, a structural transition from Form II to Form I that took place in a wide temperature range during cooling. The spectra also showed a continuous increase of the intensity of the peak at 1505 cm^−1^, which could therefore not be associated with a defined structure since the observed evolution was always detected, independent of the predominant structure (Form I or II).

### 3.5. Temperature Evolution on Heating of X-Ray Diffraction Profiles of Nylon 4 9 Samples Having Different Initial Structures

**Figure 12:**
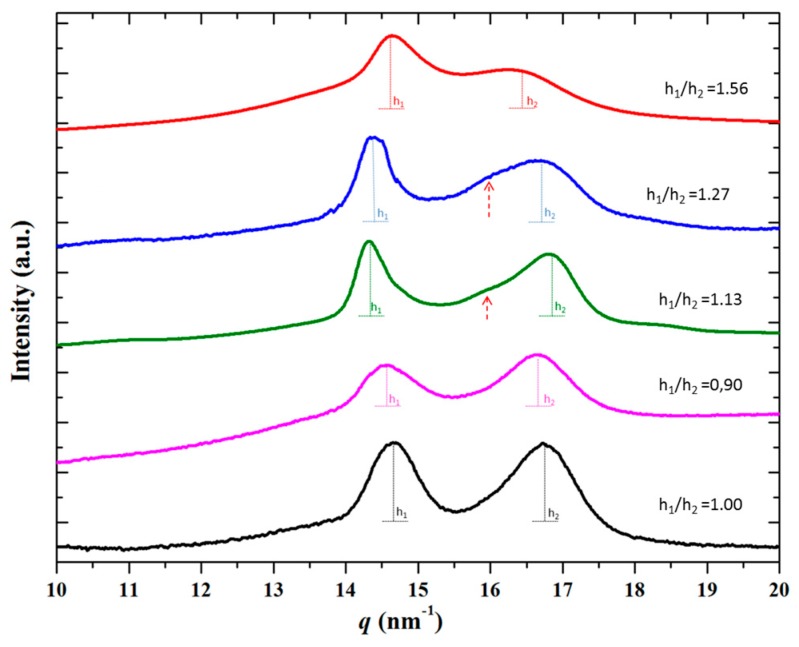
Diffraction profiles of nylon 4 9 samples taken at room temperature from down to up: as synthesized sample, solvent casting film from HFIP at a polymer concentration of 2 mg/mL, solvent casting film from formic acid at a polymer concentration of 2 mg/mL, solvent casting film from formic acid at a polymer concentration of 10 mg/mL and a melt quenched sample.

The ratio between the different crystalline forms of nylon 4 9 that can be found at room temperature logically depend on the preparation method as it was observed when the X-ray diffraction patterns of as synthesized and melt crystallized samples were compared. Figure 12 shows additional patterns of samples obtained by solvent casting from a 1,1,1,6,6,6-hexafluoroisopropanol (HFIP) solution, from formic acid solutions at different polymer concentrations and from a melt quenched sample. All samples coming from solution showed a clear peak at 0.375 nm and a second one in the 0.440–0.428 nm range, but differences on the their relative ratio (measured as h_1_/h_2_, being the numerator and the denominator the height of the peaks corresponding to the higher and lower spacings, respectively) were found. Thus, the relative intensity of the lower spacing reflection decreased at the same time that appeared a shoulder (see red arrows in Figure 12) close to 0.42 nm and related to Form II. Profiles clearly indicated that formation of Form II was favored when HFIP was changed by formic acid and when the polymer concentration in the solution increased. The spectra of the melt quenched sample were clearly different since reflections at 0.42 and 0.375 were overlapped giving rise to a broad signal with a low intensity peak (i.e., Form II was obtained in a greater ratio than in the preceding cases).

**Figure 13:**
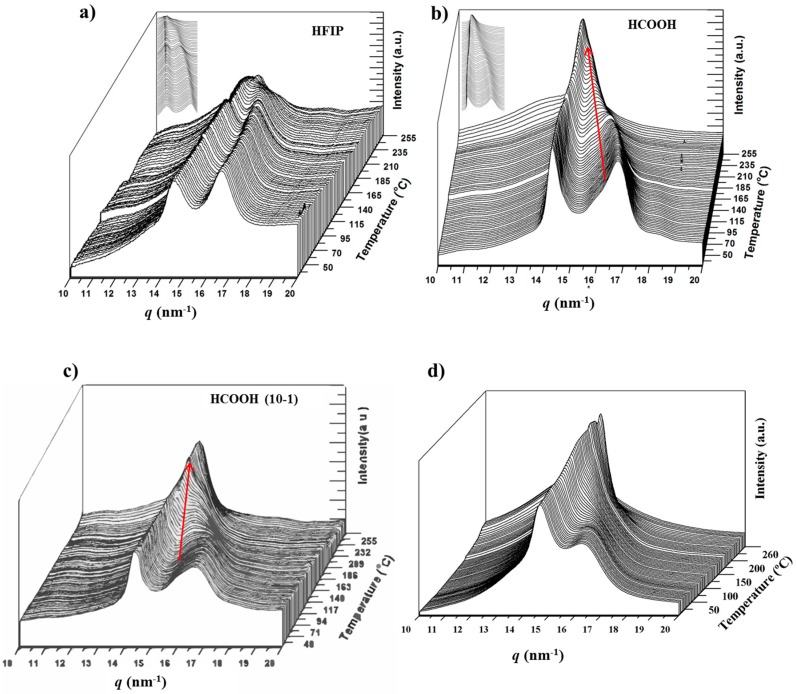
Three dimensional representation of WAXD profiles of nylon 4 9 during heating (10 °C/min) from room temperature to fusion for: (**a**) solvent casting film from HFIP at a polymer concentration of 2 mg/mL; (**b**) solvent casting film from formic acid at a polymer concentration of 2 mg/mL; (**c**) solvent casting film from formic acid at a polymer concentration of 10 mg/mL; and (**d**) melt quenched sample.

Figure 13 compares the evolution of diffraction profiles of the different solvent casting films and the melt quenched sample during a heating rate up to fusion. The most relevant feature is that all samples initially having some percentage of Form II displayed a clear transition from Form I to Form II, which appeared clearer for higher Form II contents (e.g., red arrows in the profiles of solvent casting films from formic acid at low and high polymer concentrations). This is obvious for the melt quenched sample, which is characterized by a significant content of Form II. By contrast, the solvent casting film from HIFP showed a similar behavior to that observed for the as synthesized sample. Results point out that samples having only Form I could not structurally transition to Form II, with the presence of Form III being detected only at temperatures close to fusion. More interesting is the finding that the presence of small amounts of Form II enhanced the structural transition. These became potential nuclei that favored the change in the packing arrangement.

### 3.6. Thermal Properties of Nylon 49

**Figure 14:**
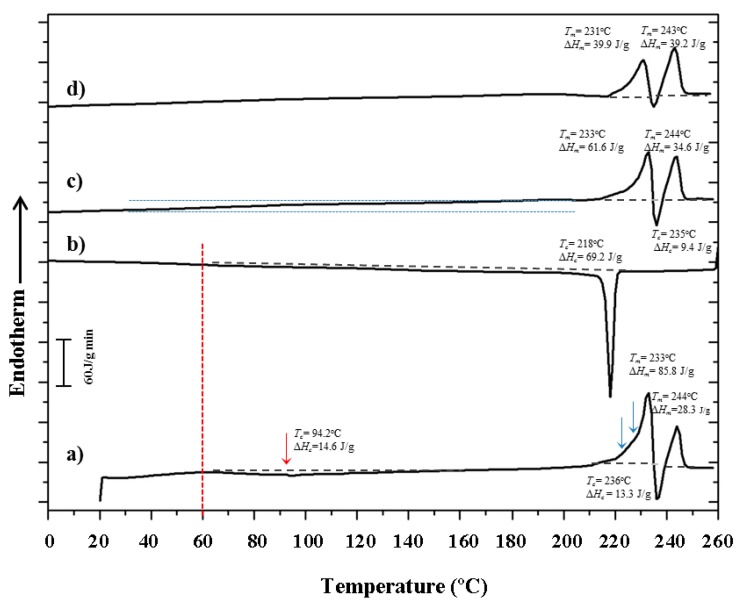
(**a**) Heating run of the as-synthesized nylon 4 9; (**b**) cooling run of nylon 4 9 from the melt state; (**c**) subsequent heating run of the melt crystallized sample; and (**d**) heating run from a nylon 4 9 melt quenched sample. Arrows indicate small endothermic and exothermic peaks.

DSC heating and cooling runs (Figure 14) revealed a complex thermal behavior of nylon 4 9, which is logically linked to the above structural features during non-isothermal treatments.

The following points should be emphasized:

(a) The first heating scan shows two clear endothermic peaks at 233 °C and 244 °C, which are related to a typical reorganization process where thin lamellae melt and recrystallize, giving rise to thicker crystals. An exothermic peak at an intermediate temperature (236 °C) is also clearly detected and confirms the existence of the indicated reorganization process. According to the synchrotron data, only the high temperature Form III should be involved in the melting and recrystallization processes.
(b) A very broad exothermic peak (red arrow) can also be detected in the first run after the glass transition temperature (50 °C according to the change of the base line) and up to 150 °C. This process fits with the cold crystallization associated with Form I that was detected in the diffraction profiles (Figure 6a and Figure 7a) during heating.
(c) The first melting peak (233 °C) observed in the first run shows two small shoulders in the low temperature region (blue arrows), which may be related to structural transitions that could involve thin and thick lamellae having the Form I structure, according to the X-ray diffraction data. Therefore, shoulders may correspond to the transition from Form I to Form III for the different lamellar populations. A similar complex process has recently been reported for nylon 6 9 [41]. However, it cannot be ruled out that these endothermic transitions were associated with fusion of Form I lamellae.
(d) The cooling scan is defined by a well-defined exothermic peak at 218 °C followed by a small and broad exotherm that extends up to a temperature close to *T_g_*. In this way, the first exotherm corresponds to crystallization into Form III, whereas the subsequent broad exotherm should be linked to the continuous structural transitions (involving Forms I and II) that were detected in the diffraction profiles.
(e) The heating scan of the melt crystallized sample showed the indicated two melting peaks at 233 °C and 244 °C. The lamellar reordering process seems to be emphasized over that observed in the first heating scan because the relative area of the second melting peak clearly increased. This is expected when thin lamellae are less stable due to a worse crystallization process, suggesting that solution crystallized samples have a higher degree of perfection than melt crystallized ones. This conclusion is also in agreement with the differences observed in the global melting enthalpy of both samples (i.e., 85.8 + 28.3 − 13.3 = 100.8 J/g and 61.6 + 34.6 − 9.4 = 86.8 J/g for solution and melt crystallized samples, respectively). The DSC scan is also characterized by a constant deviation of the base line from *T_g_* up to 200 °C, which may be due to the continuous structural transitions detected in the X-ray diffraction profiles during heating. Note also that shoulders before the first melting peak are less relevant for the melt crystallized samples, suggesting that structural transitions took mainly place during the heating process. It is highly relevant that the resultant endothermic enthalpy (86.8 J/g) associated with the processes occurring at temperatures higher than 200 °C was clearly higher than the enthalpy associated with the crystallization process performed in the previous scan (i.e., 60 J/g for the acute peak and 69.2 J/g if the broad exotherm is also considered). This suggests that shoulders of the first melting peak correspond to structural transitions and that the corresponding enthalpies are not associated with melting processes.
(f) The heating scan of the melt quenched sample revealed, again, the above features with differences in the logical decrease of global melting enthalpy (39.9 + 39.2 − 3.2 = 75.2 J/g) and the increase of relative intensity of the high temperature melting peak over the low temperature one. In summary, the sample is less crystalline and the reorganization processes become enhanced.

Equilibrium melting temperature (*T_m_*°) is a crucial parameter in determining crystal growth rate and specifically degree of supercooling (*T_m_*° − *T_c_*). The Hoffman–Weeks extrapolation [52] is a commonly accepted method of estimating the equilibrium temperature due to its simplicity and straightforward implementation, although it is subject to criticism [53] and improvements have been proposed [54]. The method is based on Equation (1), which was deduced from a combination of the well-known Gibbs–Thomson equation and secondary nucleation theory [55]. This equation relates the melting temperature, *T_m_*, of a crystal formed at a temperature *T_c_*, the equilibrium melting temperature, *T_m_°*, and the thickening coefficient, *γ*, defined as the ratio between the thickness of the grown crystal and the initial thickness of a “virgin lamella”:

*T_m_* = *T_m_°* (1 − (1/*γ*)) + *T_c_*/*γ* (1)

A straight line is obtained by plotting *T_m_* as a function of *T_c_*, with the equilibrium temperature corresponding to the intersection of this line with the *T_m_ = T_c_* line. The validity of Equation 1 implies that lamellar crystals thicken at a specific crystallization temperature, which also influences the thickening parameter.

**Figure 15:**
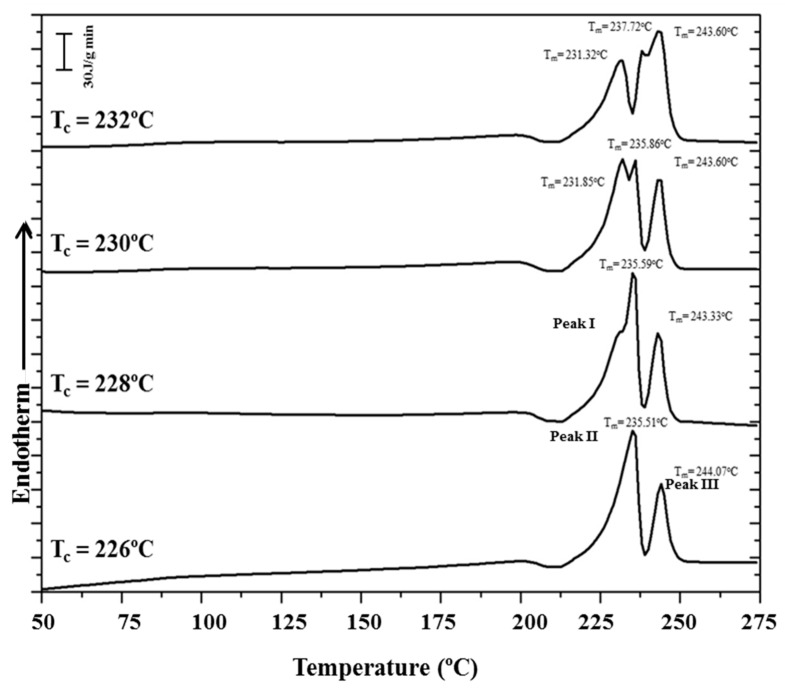
DSC traces corresponding to the heating run of samples isothermally crystallized at the indicated temperatures.

**Figure 16:**
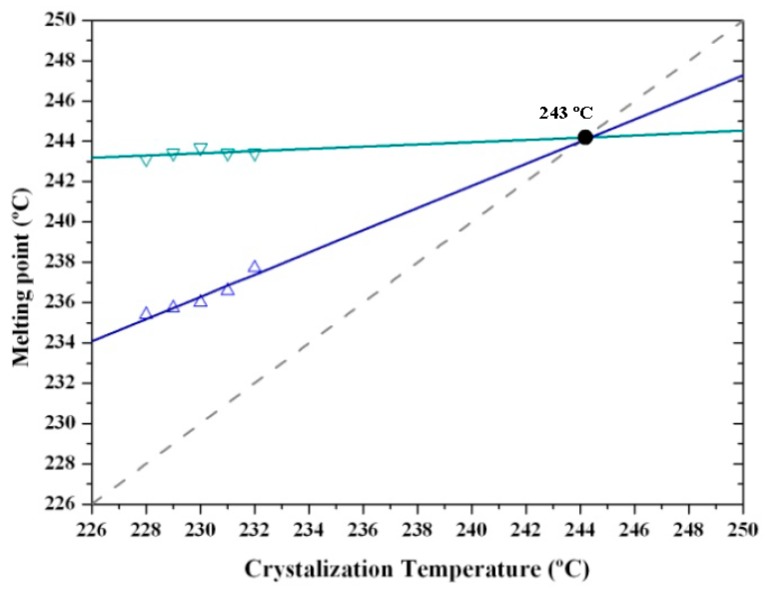
Hoffman–Weeks plot of temperatures corresponding to the observed endothermic Peaks II and III associated with the high temperature Form III versus hot crystallization temperature. An equilibrium melting temperature of 243 °C could be deduced.

Figure 15 shows the complex melting behavior of nylon 4 9 crystallized at different temperatures. At the lowest temperature (226 °C) only the two crystallization peaks associated with fusion of thin and thick lamellae of nylon 4 9 in its Form III structure were detected. The predominant melting peak (labeled as peak II) shifted to higher temperatures with the crystallization temperature, allowing the unambiguous estimation of an equilibrium melting temperature of 243 °C from the Hoffman–Weeks plot (Figure 16). The temperature of the melting peak associated with the thickest lamellae (labeled as peak III) remained practically constant and was practically identical to the estimated equilibrium melting temperature. DSC heating runs also showed a low temperature endothermic peak whose area clearly increased as the crystallization temperature did. A careful interpretation of this behavior is out of the scope of the present work because different processes are involved: isothermal crystallization at the selected temperature and non-isothermal crystallization during the subsequent heating, which could imply structural transitions between the different crystalline forms. It seems that crystallizations performed at the highest temperatures were incomplete and a greater ratio of structures corresponding to Forms II or I was found during the cooling process. Therefore, the area of the lower temperature peak (labeled as peak I) increased.

### 3.7. Spherulitic Morphologies of Nylon 4 9 Crystallized from the Melt

**Figure 17:**
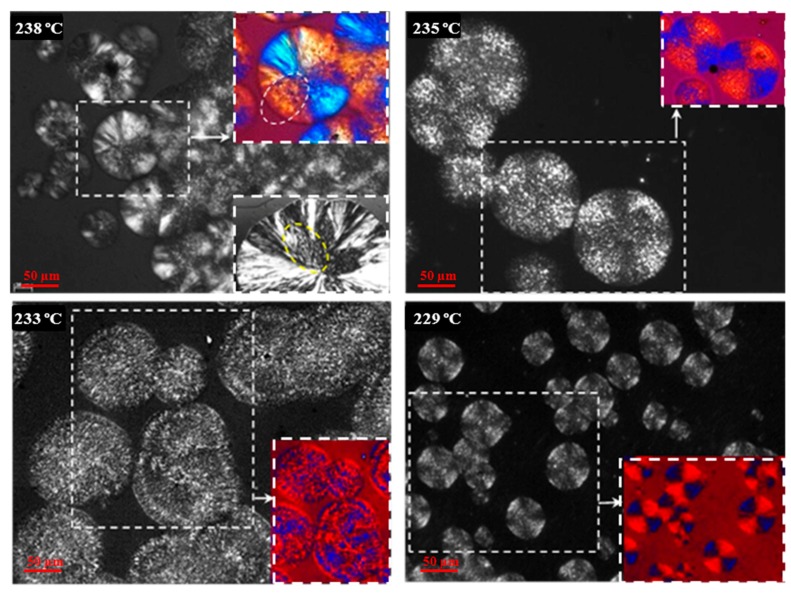
Typical spherulitic morphologies of nylon 4 9 isothermally crystallized at the indicated temperatures. Black and white inset of the micrograph taken at 238 °C reveals the complex internal structure of the obtained spherulites. Color micrographs taken with a red tin plate to determine the sign of birefringence are shown as insets for all crystallizations.

Morphology of spherulites obtained by isothermal crystallization from the melt state depended on the crystallization temperature, as shown in Figure 17 for different representative temperatures. The number of active nuclei increased with decreasing crystallization temperatures, and logically the size of spherulites decreased. Thus, diameters close to 200 µm and 25 µm were characteristic of spherulites obtained at 238 and 229 °C. Significant changes were detected despite the narrow crystallization temperature range (i.e., 9 °C). At the highest temperature, axialitic entities with complex birefringence and including domains with well-formed planar crystals (see dashed ellipsoids) were characteristic. At the lowest temperature, only ringed spherulites with a clearly negative birefringence were usually detected. At intermediate temperatures (e.g., 235 °C and 233 °C), spherulites showed a speckled texture, being detected incipient rings and a confusing birefringence at 233 °C. The predominant negative birefringence observed for nylon 4 9 spherulites contrasts with the positive sign characteristic of conventional nylons that is attained at low crystallization temperature and has been associated to a radial disposition of the single hydrogen bonding direction.

**Figure 18:**
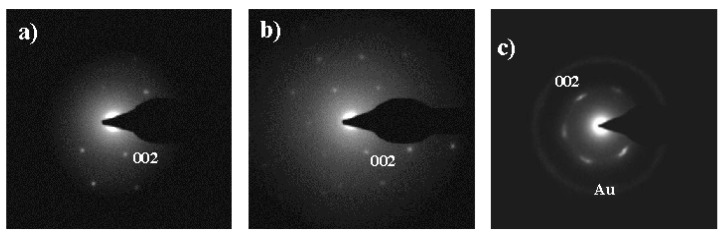
Selected-area electron diffraction pattern of nylon 4 9 spherulites crystallized at: 229 °C (**a**,**b**); and 238 °C (**c**). Patterns corresponded to the low temperature structure observed from solution crystallized samples. Symmetry is usually lost in the patterns coming from spherulites crystallized at 229 °C (**a**) as consequence of lamellar twisting, although a 2 *mm* symmetry can also hardly detected (**b**). This symmetry is more easily observed from spherulites attained at the higher temperature as a consequence of a flat-on lamellar disposition (**c**).

X-ray diffraction profiles taken in the narrow temperature interval between 238 and 229 °C corresponded to the single high temperature pseudohexagonal molecular arrangement (Form III). Thus, the differences observed in morphology and texture are not related to a change on the crystalline structure. Nevertheless, it should be considered that micrographs were taken at room temperature and consequently optical properties should be related to the predominant Form I that was achieved during cooling as deduced from synchrotron data. Electron diffraction patterns taken at room temperature of thin spherulites crystallized in the above temperature interval reflect slight variations considering the symmetry of the pattern (Figure 18). Note that lamellar twisting varied, being more difficult to obtain symmetric *hk*0 diffraction patterns at the lower temperature. Figure 18 shows the predominant electron diffraction pattern obtained from spherulites crystallized at 229 °C and 238 °C.

Kinetics of crystallization of nylon 4 9 from the melt was studied by optical microscopy. Spherulite radius grew linearly with time until impingement (not shown). Crystal growth rate (*G*) was determined in the studied temperature interval where measurable spherulites formed. 

Primary nucleation density increased dramatically with decreasing temperatures (i.e., 80 nuclei/mm^2^ and 1600 nuclei/mm^2^ were determined at 238 °C and 229 °C, respectively), and, consequently, the available temperature range for kinetic studies was highly limited. Micrographs taken during crystallization at the highest temperature showed that crystallization was mainly athermic since the number of nuclei was kept constant over time.

Secondary nucleation constants were determined by the Lauritzen–Hoffman equation [56]:

*G* = *G*_0_ × exp[−U*/(*R*(*T_c_* − *T_∞_*))] × exp[−*K_g_*/(*T_c_* (Δ*T*) *f*)] (2)

where *G*_0_ is the constant pre-exponential factor, U* is the activation energy characteristic of the transport of crystallizing segments across the liquid-crystal interface, *T_∞_* is the temperature below which such motion ceases, *R* is the gas constant, *K_g_* is the corresponding secondary nucleation constant, ΔT is the degree of supercooling measured as *T_m_* − *T_c_* (where *T_m_* is the equilibrium melting temperature and *T_c_* is the crystallization temperature), and *f* is a correction factor accounting for the variation in the bulk melting enthalpy per unit volume with temperature (*f* = 2*T_c_*/*T_m_* + *T_c_*).

**Figure 19:**
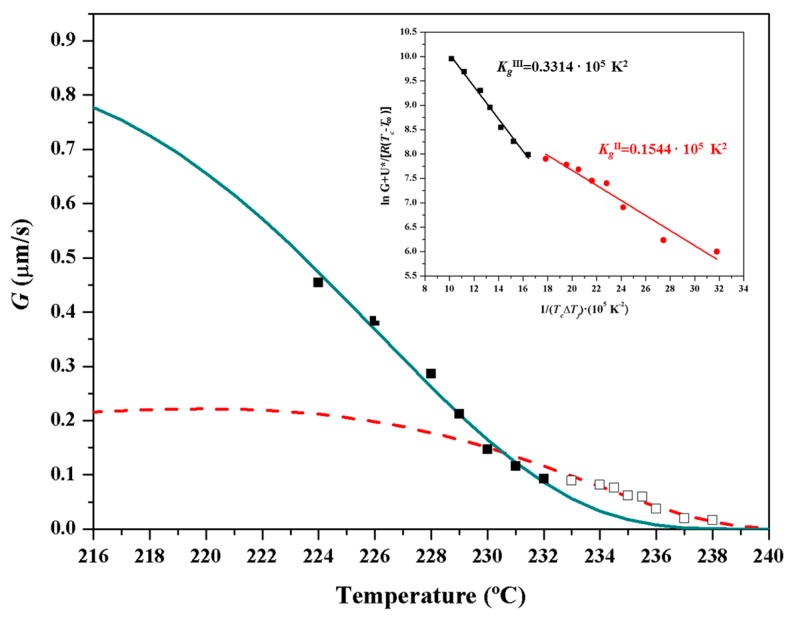
Temperature dependence of crystal growth rate (solid green line for Regime III and dashed red line for Regime II) determined by Lauritzen–Hoffman equation and using the best fit parameters deduced for the two crystallization regimes. Experimental crystal growth rates and indicated by the square symbols. The inset shows the plot of ln *G* + U*/*R*(*T_c_* − *T_∞_*) versus 1/*Tc* (Δ*T*) *f* to determine the *Kg* secondary nucleation parameters.

The inset of Figure 19 shows the linear plots obtained using U* and *T_∞_* parameters of 1600 cal/mol and *T_g_* − 35 K, respectively. It is clear that two crystallization regimes defined by secondary nucleation constants of 0.33 × 10^5^ K^2^ and 0.15 × 10^5^ K^2^ fit the experimental data. Regimes III and II could be assumed since the experimental ratio between slopes (2.2) was close to the theoretical *K_g_^III^*/*K_g_^II^* value of 2. Figure 19 also corroborates that the two bell-shaped curves calculated by the Lauritzen–Hoffman equations fit well with the experimental spherulitic growth data.

## 4. Conclusions

Nylon 4 9 crystallized from diluted glycerin solutions giving rise to lamellar single crystals which morphology varied according to the crystallization temperature. Specifically, regular rhombic crystals were attained at 120 °C, while aggregates or irregular and elongated crystals were characteristics of lower and higher temperatures, respectively. Electron diffraction patterns showed a 2 mm symmetry and pointed out a single structure (Form I) characterized by a disposition of hydrogen bonds along two planar orientations as reported for other even–odd nylons previously studied. 

Nylon 4 9 isothermally crystallized from the melt giving rise to spherulites with a texture that clearly varied in a very narrow temperature interval (i.e., from 238 °C to 229 °C). Flat-on and edge-on lamellar dispositions were detected at the highest and the lowest crystallization temperatures, changing the morphology from axialites to ringed spherulites and the formation of speckled spherulites at intermediate temperatures. Electron diffraction patterns indicate that all these spherulites predominantly had the Form I structure when were observed at room temperature. At this temperature, the birefringence sign was negative in contrast with the positive sign detected for conventional even–even polyamides. Crystallization kinetic analysis indicated that two crystallization regimes were characteristic of nylon 4 9, being the involved temperatures in agreement with the observed morphologic changes.

Nylon 4 9 showed complex transitions during heating and cooling processes that depended on the way as the sample was prepared. Before melting, nylon 4 9 achieved a typical pseudohexagonal arrangement (Form III) that was consequence of the named Brill transition. A new polymorphic structure (II) appeared during crystallization from the melt as well as from crystallization from some specific solvents. Temperature evolution of diffraction patterns during heating processes was clearly dependent on the presence of Form II. Thus, profiles indicate a direct transition from Form I to Form III or from Form I to Form II when traces of the intermediate structure (Form II) were absent or present, respectively. The complex behavior was also supported by calorimetric and spectroscopic data, being of interest for the understanding/racionalization of the high structural variability described for nylons in general and even the differences detected between even–odd nylons in particular.
